# Development of fermented sea buckthorn (*Hippophae rhamnoides* L.) juice and investigation of its antioxidant and antimicrobial activity

**DOI:** 10.3389/fnut.2023.1120748

**Published:** 2023-01-20

**Authors:** Xiaolu Liu, Mingshan Lv, Ruxianguli Maimaitiyiming, Keping Chen, Nuersiman Tuerhong, Jiangyong Yang, Aihemaitijiang Aihaiti, Liang Wang

**Affiliations:** ^1^School of Life Sciences and Technology, Xinjiang University, Ürümqi, China; ^2^Xinjiang Huize Food Limited Liability Company, Ürümqi, China

**Keywords:** sea buckthorn (*Hippophae rhamnoides* L.), fermentation, response surface methodology, antioxidant activity, antimicrobial activity

## Abstract

Sea buckthorn (*Hippophae rhamnoides* L.) is an edible and medicinal plant species. However, due to its sour taste, it is not readily accepted by consumers. To overcome this, fermentation can be used to change its flavor profile. In this study, we used response surface methodology (RSM) to determine the best process for producing fermented sea buckthorn juice (FSBJ) using probiotics. The biological enzyme activity and total flavonoid content (TFC) of sea buckthorn juice (SBJ) increased after fermentation. When the number of bacteria inoculated was 4.08 × 10^6^ CFU/mL and the inoculation ratio was 30% *Z. mobilis*, 5% *L. casei*, 13.75% *L. plantarum*, 31.25% *P. acidilactici*, 12.5% *L. animalis*, and 7.5% *P. pentosaceus*, the amount of sugar was 2.98% (w/v) after 20 h of fermentation at 37°C, and the superoxide dismutase (SOD) activity reached 725.44 U/mL, and the TFC reached 2.38 mg/mL. FSBJ demonstrated strong antimicrobial activity against *Escherichia coli*, *Staphylococcus aureus* and *Botrytis cinerea*. Then, to investigate the antioxidant capacity of FSBJ, we used H_2_O_2_ to induce oxidative stress in C2C12 cells and assessed the protection conferred by FSBJ to damaged cells. It was discovered that after 24 h of treatment with FSBJ, not only was there an increase in the activities of intracellular SOD and glutathione peroxidase (GSH-Px), but also a reduction in reactive oxygen species (ROS) content, catalase (CAT) activity, and malondialdehyde (MDA) content. This research lays the theoretical groundwork and provides reference materials for the improved fermentation of sea buckthorn and demonstrates its resulting antioxidant effect.

## 1. Introduction

Sea buckthorn (*Hippophae rhamnoides* L.) is a medicinal and food homologous material in China that is used in traditional Chinese medicine. Records reveal that sea buckthorn has been cultivated and consumed for medicinal purposes in China for over 1,000 years. Due to its excellent medicinal and nutritional properties, sea buckthorn is widely used to treat and prevent Alzheimer’s disease (1), cardiovascular disease (2), gastric ulcers (3), cancer (4), and skin problems (5). These applications of sea buckthorn are related to the rich active biological substances that are present in its berries, such as flavonoids (6), polysaccharides (7), polyphenols (8), organic acids (9), vitamins (10), amino acids (11), and other active substances (12). Although sea buckthorn berries are nutritious, their taste is sour and astringent, thereby rendering the plant unpleasant to eat raw. Moreover, the shelf life of sea buckthorn is very short, as it is highly susceptible to mechanical damage and fungal infection during its storage and transportation. Therefore, there is value in researching and developing a processing method for sea buckthorn fruit that is cheap, beneficial for improving its flavor profile, and adds to its nutritional value.

Fermentation is a food processing method that converts complex organic compounds into simpler ones through the growth and metabolic activity of probiotics, such as lactic acid bacteria and yeast (13). Fruit juice is rich in sugars, organic acids, amino acids, vitamins, minerals, and so on, which can provide the necessary carbon sources, nitrogen sources, inorganic salts, and water for the growth of probiotics (14). Many studies have shown that probiotic fermentation allows some of the original nutrients in the raw materials to be retained, while others are metabolized to produce a range of new bioactive substances. Probiotics can produce a series of aromatic substances such as alcohols, esters, and terpenes by hydrolyzing the polyphenols, glucosinolates, polypeptides, and metabolic enzymes that are naturally present in fruit and vegetable juices (15). The metabolic processes employed by probiotics can also reduce the generation of undesirable compounds (16). The pharmacological activities of many bioactive substances are enhanced under the action of probiotic biotransformation (17). Fuente et al. (15) studied the influence of lactic acid bacteria on the physicochemical indexes of orange juice and found that fermented orange juice had higher total polyphenol content and antioxidant capacity (18). Probiotic fermentation can produce a variety of antagonistic metabolites, such as antifungal compounds and bacteriocins, which can inhibit the growth of harmful microorganisms during storage (19). Therefore, probiotic fermentation may represent an economical means that can enhance the nutritional properties, improve the content and activity of bioactive compounds, modify the flavor properties, and increase the shelf life of various products. Nonetheless, when the process of fermentation is not tightly controlled, there will always be slight variations in the active ingredients and flavor substances produced from batch to batch, regardless of whether a similar material is fermented, and these variations will consequently influence the efficacy and quality of the finally product (20). Accordingly, screening for the most suitable strains and determining the optimal processing conditions to enhance the nutritional and sensory value of fermented sea buckthorn juice becomes critical.

Response surface methodology (RSM) is an experimental optimization method that is widely employed in food processing. It can be used to effectively and quickly determine the optimal conditions for a multi-factor system by using a rational experimental design that allows for the comprehensive study of a given experiment in the most economical way. Box-Behnken design (BBD) is a commonly experimental design method used for RSM. It can provide a multi-factor, three-level experimental design, using multivariate quadratic equations to fit the functional relationships between factors and response values, and seeking the optimal process parameters through the analysis of regression equations.

In this paper, FSBJ was developed by conducting fermentation using six kinds of probiotics. Firstly, six strains were chosen by using a single-factor test based on the criteria of having a high superoxide dismutase (SOD) production capacity, producing a high total flavonoid content (TFC), and producing a flavor profile deemed desirable by sensory evaluation. The optimal percentage of inoculum was also predicted by a unified design test. Then, based on preliminary experiments, the fermentation process was optimized using SOD and the TFC as indicators. The antibacterial activity and antifungal activity of FSBJ were studied. In addition, the resulting FSBJ products were assessed for their ability to protect cells damaged by oxidative stress induced by hydrogen peroxide, whereby the differential effects of SBJ and FSBJ on the cellular reactive oxygen species (ROS) content of C2C12 cells were detected by flow cytometry. To investigate the mechanism by which fermented sea buckthorn juice reduces oxidative stress-induced damage, the malondialdehyde (MDA) content, SOD activity, catalase (CAT) activity, and glutathione peroxidase (GSH-Px) activity in the cells were measured.

## 2. Materials and methods

### 2.1. Materials

Sea buckthorn berries were provided by Xinjiang Huize Food Co., Ltd, Xinjiang, China. Upon their reception, they were immediately stored in a freezer at −18°C. The DeMan, Rogosa, and Sharpe (MRS), Malt Extract Broth (MEB), and Luria-Bertani (LB) culture medium were purchased from Basebio Biotechnology Co., Ltd. (Hangzhou, China). Trypsin-EDTA (0.05%) and Dulbecco’s Modified Eagle’s Medium (DMEM) were purchased from Gibco Inc. (Shanghai, China). Dimethyl sulfoxide (DMSO) was obtained from Solarbio Biotechnology Co., Ltd. (Shanghai, China). Pectinase, cellulase, and hemicellulase were purchased from Yuanye Co., Ltd. (Wuhan, China). The assay kits used to measure SOD, GSH-Px, MDA, and CAT were provided by the Jiancheng Biotechnology Institute (Nanjing, China). Cell Counting Kit-8 (CCK-8) assay kits were provided by Biosharp Biotechnology Co., Ltd. (Shanghai, China). Fetal bovine serum (FBS), and phosphate-buffered saline (PBS) were provided by BI Biotechnology Co., Ltd. (Shanghai, China). The anaerobic bacteria used in this study (*Lactobacillus paracasei*, *Lactobacillus casei*, *Pediococcus pentosaceus*, *Pediococcus acidilactici*, *Zymomonas mobilis*, *Lactobacillus animalis*, *Lactobacillus fermentum*, *Lactobacillus delbrueckii*, *Lactobacillus reuteri*, *Lactobacillus acidophilus*, *Lactobacillus rhamnosus*, and *Lactobacillus plantarum*) were obtained from the China General Microbiological Culture Collection Center (Beijing, China). C2C12 cells, *Escherichia coli*, *Staphylococcus aureus* and *Botrytis cinerea* were obtained from BNCC Technology Co., Ltd. (Beijing, China).

### 2.2. Preparation and fermentation of sea buckthorn juice

First, the fruit was washed and squeezed into a homogenate by an electric juice extractor. The homogenate was then placed in 90°C water for 15 min to deactivate the enzymes and microorganisms. Then, the homogenate was cooled to room temperature and added with 0.4% (w/v) pectinase, 0.2% (w/v) cellulase, 0.2% (w/v) hemicellulase, and 3% (w/v) sugar before undergoing hydrolysis at 55°C for 3 h. The juice was sterilized at 80°C for 30 min after enzymatic hydrolysis and cooled to room temperature being inoculated with the fermentation strains.

### 2.3. Screening of dominant strains and determination of their optimal proportions

SBJ was inoculated with 12 different strains individually before being incubated at 37°C for 20 h. At the end of the fermentation period, the SOD activity, TFC, and sensory scores of the FSBJ products were measured, and the dominant strains were chosen on the basis of the results. To obtain the optimal percentages of the six bacteria in the inoculum, a uniform design table with six factors and 10 levels was used (Table 1). Then, the regression equation was analyzed using SPSS 26 software and finally the results were calculated using Excel.

**TABLE 1:** Uniform design factor level table.

Strains	Code	Run number									
		1	2	3	4	5	6	7	8	9	10
*Zymomonas Mobilis*	X1 (%)	15	16	17	18	19	20	21	22	23	24
*Lactobacillus Casei*	X2 (%)	12	5	10	8	6	13	7	4	9	11
*Lactobacillus Plantarum*	X3 (%)	5	11	8	4	2	9	7	6	10	3
*Pediococcus Acidilactici*	X4 (%)	30	28	33	25	34	27	26	31	32	29
*Lactobacillus animalis*	X5 (%)	12	16	19	10	15	14	18	13	11	17
*Pediococcus Pentosaceus*	X6 (%)	7	10	13	12	9	14	6	15	8	11

### 2.4. Optimization of the fermentation process

To investigate the effects and ideal proportion of fermentation temperature (27, 32, 37, 42, or 47°C), added sugar (0, 1, 2, 3, or 4%), fermentation time (16, 18, 20, 22, or 24 h), and inoculum amount (1, 2, 4, 8, or 16 × 10^6^ CFU/mL) on the SOD activity and TFC values of FSBJ, single-factor experiments were conducted. Then, a Box–Behnken test was designed with the fermentation temperature(A), fermentation time (B), inoculum amount (C), and added sugar amount (D) as independent variables, with SOD activity (Y_1_) and the TFC (Y_2_) as the response values. Each factor was assigned three levels (−1, 0, 1), and the design included five central points for a total of 29 experimental combinations. The table of factors and levels of the BBD is shown in Table 2. The results of the response surface experiments are shown in Table 3.

**TABLE 2:** Factors and levels of the experiment.

Factors	Levels		
	−1	0	1
Fermentation temperature (°C)	32	37	42
Fermentation time (h)	18	20	22
Inoculum volume (*10^6^ CFU/mL)	2.0	4.0	6.0
Added sugar amount (%)	2.5	3.0	3.5

**TABLE 3:** The experiments and results of RSM for the SBJ fermentation process.

No.	Factors				Response	
	Temperature (°C)	Time (h)	Inoculation amount (*10^6^CFU/mL)	Added sugar amount (%)	SOD activity (U/mL)	TFC (mg/mL)
1	37	18	2	3.0	590.14 ± 2.90	2.29 ± 0.03
2	37	20	4	3.0	654.12 ± 7.23	2.50 ± 0.04
3	37	22	4	2.5	600.88 ± 8.29	2.35 ± 0.02
4	42	20	4	3.5	580.32 ± 10.67	2.39 ± 0.06
5	42	20	4	2.5	595.79 ± 9.34	2.37 ± 0.02
6	32	20	2	3.0	610.46 ± 7.55	2.26 ± 0.05
7	32	20	4	2.5	599.57 ± 5.43	2.27 ± 0.02
8	37	22	6	3.0	588.02 ± 9.42	2.35 ± 0.04
9	32	18	4	3.0	589.08 ± 11.34	2.25 ± 0.05
10	37	20	2	3.5	576.33 ± 8.43	2.38 ± 0.01
11	42	22	4	3.0	586.97 ± 7.23	2.29 ± 0.02
12	37	22	4	3.5	605.94 ± 8.10	2.43 ± 0.07
13	37	20	4	3.0	655.55 ± 6.89	2.51 ± 0.01
14	37	18	4	2.5	614.50 ± 4.90	2.40 ± 0.02
15	42	18	4	3.0	602.03 ± 13.99	2.34 ± 0.01
16	32	20	6	3.0	579.75 ± 11.39	2.36 ± 0.00
17	37	20	4	3.0	648.08 ± 8.44	2.49 ± 0.05
18	42	20	6	3.0	587.36 ± 5.92	2.33 ± 0.00
19	37	22	2	3.0	617.19 ± 6.45	2.43 ± 0.00
20	32	22	4	3.0	618.83 ± 8.78	2.28 ± 0.02
21	37	18	4	3.5	579.48 ± 10.23	2.37 ± 0.01
22	37	18	6	3.0	598.37 ± 8.66	2.44 ± 0.05
23	37	20	4	3.0	662.48 ± 12.43	2.48 ± 0.03
24	37	20	2	2.5	604.36 ± 13.31	2.35 ± 0.03
25	37	20	4	3.0	646.02 ± 8.88	2.46 ± 0.03
26	37	20	6	3.5	579.06 ± 5.54	2.50 ± 0.02
27	42	20	2	3.0	593.09 ± 9.57	2.38 ± 0.00
28	37	20	6	2.5	584.36 ± 10.43	2.39 ± 0.01
29	32	20	4	3.5	585.43 ± 2.56	2.32 ± 0.00

### 2.5. Measurement of physicochemical indicators

**FIGURE 1:**
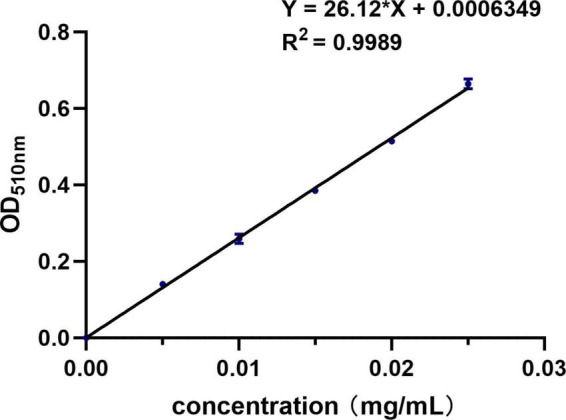
Standard curve of the NaNO_2_-Al(NO_3_)_3_-NaOH coloring method.

The activity of SOD was determined by corresponding kit’s instructions. The TFC was determined by the method reported by Yan (20) and calculated according to the standard curve equation (Figure 1). Briefly, 1 mL of sample was diluted 10 times into a 10 ml volumetric flask before 300 μL of 50 mg/mL NaNO_2_ was added and left for 6 min. Then, 300 μL of 100 mg/mL Al (NO_3_)_3_ was added and left for another 6 min. Finally, 4 mL of 1 mol/L NaOH was added and the volume was fixed with ethanol. The absorbance was finally measured at 510 nm.

Sensory evaluation was carried out to assess the sensory properties of liquid fermented juice (21). FSBJ produced under different conditions were poured into the coded transparent cups. Ten assessors who were trained in sensory assessment rated the appearance, flavor, taste, acceptability, and their hypothetical purchasing intention of the FSBJ products, and the total score was 110 points.

### 2.6. Assay of C2C12 cell viability

C2C12 cells were cultured in DMEM complete medium at 37°C and 5% CO2 was injected into the incubator. The old medium was replaced by fresh medium every day. Cell viability was measured using the CCK-8 kit. C2C12 cells were seeded in 96-well plates with cell concentration at 2.5 × 10^4^ cells per well and then cultured for 24 h. The control and experimental groups were set with six parallel groups for each. Blank control wells had only medium added and were set up at the same time. When the cells covered 70-80% of the pore bottom, medium containing different doses of either SBJ (0, 0.5, 1, 2, 4, or 8 mg/mL) or FSBJ (0, 0.5, 1, 2, 4, or 8 mg/mL) was added and treated for 24 h. The culture medium was then removed. After adding 100 μL of DMEM medium and 10 μL of CCK-8 to each well, the culture was continued to observe for the color changes. Finally, the cell optical density (OD) was measured at 450 nm by using an ELISA reader.

### 2.7. Establishing the cellular oxidative stress model

C2C12 cells were treated with H_2_O_2_ (oxidative stress model group) and a control group was set up without H_2_O_2_ treatment. The cells were treated with different concentrations of H_2_O_2_ (0, 125, 250, 500, 1,000, or 2,000 μmol/L) for 12, 24, or 48 h. The optimal H_2_O_2_ concentration and treatment time (500 μmol/L, 24 h) were then obtained, after which, the cell viability was evaluated by the CCK-8 assay.

The above oxidative stress cell model was used to investigate the antioxidant activity of FSBJ. Firstly, C2C12 cells were seeded in 96-well plates and divided into the control, H_2_O_2_, H_2_O_2_ + SBJ (0.5, 1, or 2 mg/mL) and H_2_O_2_ + FSBJ (0.5, 1, or 2 mg/mL) groups. Then the culture medium was discarded and the 96-well plate was treated with the H_2_O_2_ to induce oxidative stress in its cells.

### 2.8. Determination of antioxidant activity

The cells were detached and seeded into 6-well plates. The control, H_2_O_2_ model, SBJ treatment, and FSBJ treatment groups were set, with three wells in each group. After undergoing the respective treatments described in section 2.7, use pancreatic enzymes to detachment and collect the cells. The activities of SOD, MDA and GSH-Px of the cells were detected by the corresponding kits.

C2C12 cells were seeded into 6-well plates with 6 × 10^5^ cells per well and then adherent cultured. After being administered the respective treatments listed above, the cells were washed with PBS and fluorescently stained with DCFHDA according to the kit instructions. Then, flow cytometry was conducted to determine the intracellular ROS content.

### 2.9. Antimicrobial activity

*Staphylococcus Aureus* and *E. coli* were inoculated into the LB broth and cultured until the density reached 10^6^ CFU/ml, the culture conditions were 37°C for 24 h. A 96 shallow wells plate assay was used to evaluate the antibacterial activity of FSBJ. The contents of bacterial suspension and FSBJ in the pore plate were 100 μL respectively in the 96 shallow well plate (22), and were cultured in a 37°C incubator for 24 h. The appropriate density of the culture broth was measured at 600 nm (OD_600_) with a microplate reader at 0 and 24 h and recorded as A_*S0*_ and A_*S24*_, respectively. A control was also maintained by mixing the bacterial suspension with LB broth instead of FSBJ, and its absorbance results were recorded as A_*C0*_ and A_*C24*_, respectively. The antibacterial efficiency was calculated according to the formula:

$$\mathrm{Inhibition}\%=\frac{\left({\text{A}}_{\text{C}\,24}-A_{\text{C}\,0}\right)-\left({\text{A}}_{\text{S}\,24}-A_{\text{S}\,0}\right)}{{\text{A}}_{\text{C}\,24}-A_{\text{C}\,0}}\times 100$$

The fungi *B. cinerea* was seeded in MEB and incubated at 26 °C for 5 days, before being diluted into a suspension of 10^6^ conidia/mL. In a 96 shallow well plate, 100 μL of MEB (10^6^ conidia/mL) was mixed with 100 μL of FSBJ (22), and incubated at 26°C for 72 h. The optical density of the culture broth was subsequently measured at 600 nm at 0 and 72 h and recorded as A_*S0*_ and A_*S72*_, respectively. A control was also maintained by mixing conidia suspension with MEB broth instead of FSBJ, and the absorbance results were recorded as A_*C0*_ and A_*C72*_, respectively. The antifungal efficiency was calculated according to the formula:

$$\mathrm{Inhibition}\%=\frac{\left({\text{A}}_{\text{C}\,72}-A_{\text{C}\,0}\right)-\left({\text{A}}_{\text{S}\,72}-A_{\text{S}\,0}\right)}{{\text{A}}_{\text{C}\,72}-A_{\text{C}\,0}}\times 100$$

### 2.10. Statistical analysis

Experimental data obtained from three parallel experiments were expressed as the mean ± standard deviation or average values. Significance analysis of parallel data and between-group data was analyzed using SPSS 26. All figures were drawn using GraphPad Prism v8.0.2. RSM data optimization was performed using the Design-Expert 13 software (Table 3). RSM with a BBD was used to process the experimental data. The statistical significance of the items in the regression equation was tested. Significant terms in the model were found by performing an analysis of variance (ANOVA) for each response (Table 4).

**TABLE 4:** The analysis of variance of FSBJ by SOD activity and the TFC.

	SOD activity (U/mL)		TFC (mg/mL)	
Source	*F*-value	*P*-value	*F*-value	*P*-value
Model	59.75	<0.0001**	23.91	<0.0001**
A-Temperature	5.50	0.0343*	22.98	0.0003**
B-Fermentation time	7.62	0.0153*	0.2837	0.6026
C-Inoculum amount	21.71	0.0004**	13.90	0.0022**
D-Amount of added sugar	33.62	<0.0001**	11.99	0.0038**
AB	23.47	0.0003**	3.40	0.0863
AC	7.29	0.0172*	11.97	0.0038**
AD	0.0207	0.8877	0.4787	0.5003
BC	16.35	0.0012**	28.14	0.0001**
BD	18.77	0.0007**	6.44	0.0237*
CD	6.04	0.0277*	3.40	0.0863
A^2^	273.12	<0.0001**	195.44	<0.0001**
B^2^	149.01	<0.0001**	70.55	<0.0001**
C^2^	320.99	<0.0001**	23.77	0.0002**
D^2^	327.11	<0.0001**	15.95	0.0013**
Lack of Fit	0.3036	0.9425^ns^	1.38	0.4057^ns^
R^2^	0.9835	-	0.9598	-
Adj. R^2^	0.9671	-	0.9197	-
Pred. R^2^	0.9445	-	0.8066	-
Adeq. Precision	22.9864	-	16.1196	-
C.V.%	0.7650	-	0.9117	-

*p < 0.05, **p < 0.01.

## 3. Results

### 3.1. Screening of dominant strains and determination of their optimal proportions

Different strains play different roles in the fermentation process, and the features of each strain must be considered when selecting a strain that is suitable for sea buckthorn juice as the fermentation medium. According to previous research, blended strains exert more favorable impacts on fermentation than single strains (23). With the mutualistic symbiotic relationships that occur between microbes, each strain serves a specific purpose, and by this mechanism, mixed-bacteria fermentation can compensate for the shortcomings of single-strain fermentation (24). Fruit juice that is fermented by mixed strains has a flavor and nutritional value that cannot be obtained by only one strain (25). So, from a pool of 12 strains, this study chose the dominant strains to use for the ensuing mixed fermentation. The results of single bacteria fermentation are shown in Table 5.

**TABLE 5:** The characteristics of SBJ fermented by twelve separate strains.

Strains	pH	TSS	SOD Activity (U/mL)	TFC (mg/mL)	Sensory evaluation (Score)	Bacterial density (× 10^8^CFU/mL)
Unfermented	3.15 ± 0.00^bc^	14.1 ± 0.08^ab^	548.91 ± 0.00^i^	2.10 ± 0.00^ef^	85.00 ± 0.82^e^	/
*Lactobacillus reuteri*	3.10 ± 0.01^fg^	13.8 ± 0.00^cd^	564.78 ± 4.62^i^	2.04 ± 0.04^f^	82.26 ± 1.03^g^	2.67 ± 0.42^def^
*Lactobacillus Casei*	3.17 ± 0.01^ab^	13.6 ± 0.26^de^	584.19 ± 5.65^h^	2.33 ± 0.05^a^	92.13 ± 0.30^a^	2.13 ± 0.19^ef^
*Lactobacillus paracasei*	3.12 ± 0.00^defg^	13.5 ± 0.08^e^	591.18 ± 10.90^gh^	2.21 ± 0.02^cd^	84.46 ± 1.48^ef^	1.76 ± 0.42^f^
*Lactobacillus rhamnosus*	3.14 ± 0.01^cde^	13.9 ± 0.19^bc^	601.51 ± 2.56^fg^	2.10 ± 0.04^ef^	86.03 ± 1.18^de^	2.83 ± 0.51^def^
*Lactobacillus delbrueckii*	3.10 ± 0.00^fg^	13.6 ± 0.14^de^	603.84 ± 8.98^ef^	2.21 ± 0.01^c^	85.43 ± 1.50^e^	5.32 ± 0.45^ab^
*Lactobacillus acidophilus*	3.09 ± 0.01^g^	13.6 ± 0.12^de^	606.63 ± 7.59^ef^	2.25 ± 0.03^bc^	82.46 ± 0.53^fg^	4.96 ± 0.85^ab^
*Zymomonas mobilis*	3.14 ± 0.02cd	13.6 ± 0.17de	608.21 ± 13.31e	2.32 ± 0.01ab	88.80 ± 0.64c	3.70 ± 0.33^cd^
*Pediococcus acidilactici*	3.12 ± 0.01^def^	14.1 ± 0.14^ab^	625.53 ± 8.16^de^	2.31 ± 0.07^ab^	82.89 ± 1.42^fg^	5.77 ± 0.16^a^
*Lactobacillus fermentum*	3.12 ± 0.02^def^	14.3 ± 0.08^a^	634.76 ± 4.56^cd^	2.18 ± 0.05^cd^	82.46 ± 0.87^fg^	2.85 ± 0.43^de^
*Pediococcus pentosaceus*	3.11 ± 0.02^efg^	14.1 ± 0.09^abc^	641.48 ± 5.34^bc^	2.13 ± 0.05^de^	87.79 ± 0.65^cd^	4.38 ± 0.97^bc^
*Lactobacillus animalis*	3.15 ± 0.01^bc^	13.6 ± 0.14^de^	652.35 ± 8.98^b^	2.22 ± 0.05^c^	89.76 ± 0.67^bc^	5.83 ± 0.33^a^
*Lactobacillus plantarum*	3.18 ± 0.02^a^	13.6 ± 0.14^de^	676.63 ± 6.00^a^	2.20 ± 0.00^cd^	91.03 ± 1.19^ab^	3.18 ± 0.57^de^

Different letters in a column indicate significant differences among groups (*p* < 0.05).

After fermentation, the SOD activity of each group increased, and the bacteria that resulted in the highest SOD values were *P. pentosaceus*, *L. animalis*, and *L. plantarum*. Compared to unfermented sea buckthorn juice, they increased the SOD activity to 92.57 U/mL, 103.44 U/mL, and 127.72 U/mL, respectively. During the process of fruit juice fermentation, lactic acid bacteria can produce SOD, but their ability to do so may be related to the characteristics and adaptability of the particular strain. The amounts of TFC produced by the inoculating groups of *L. casei*, *Z. mobilis*, and *P. acidilactici* were significantly higher than that measured in the non-fermented group, which was consistent with the findings of other studies (26). This could be due either to the conversion of bound flavonoids to free flavonoids or to the conversion of polyphenols to flavonoids during fermentation, which yields an increased flavonoid content. Sensory evaluation has a strong influence on the consumer’s decision to purchase a product. Sensory scores are affected by the proportion of acidic to sweet as well as various flavor substances. The production of organic acids during fermentation reduces the pH value, and such high acidity destroys the sweet-acidity balance of the fermented juice, thereby resulting in an unfavorable taste. The sensory scores of the groups inoculated with *L. casei*, *L. plantarum*, and *L. animalis* were higher, indicating that these three bacteria are capable of producing more pleasant flavor substances. Explanations for these differences could include differences in strain growth rates, optimal growth environments, and carbon source utilization.

In summary, three optimal bacteria were chosen for each of these three factors based on high TFC, SOD activity, and sensory scores, as long as the bacteria did not have a bad influence on others factors. Finally, *L. casei*, *Z. mobilis*, *P. acidilactici*, *P. pentosaceus*, *L. plantarum*, and *L. animalis* were finally chosen as the optimal fermentation strains to ferment sea buckthorn juice. Uniform design of experiments was used to determine the most appropriate percentage of the mixed strains, and the results are shown in Table 6. The quadratic polynomial stepwise regression equation with SOD activity as the dependent variable is as follows:

Y=565.826+626.22X+12308.946XX1-3726.036X1X6-1550.19XX4-5477.368X2X+41022.014X25

**TABLE 6:** Results of the uniform design experiments.

Name	Code	Run number									
		1	2	3	4	5	6	7	8	9	10
*Zymomonas Mobilis*	X_1_ (%)	15	16	17	18	19	20	21	22	23	24
*Lactobacillus Casei*	X_2_ (%)	12	5	10	8	6	13	7	4	9	11
*Lactobacillus Plantarum*	X_3_ (%)	5	11	8	4	2	9	7	6	10	3
*Pediococcus Acidilactici*	X_4_ (%)	30	28	33	25	34	27	26	31	32	29
*Lactobacillus animalis*	X_5_ (%)	12	16	19	10	15	14	18	13	11	17
*Pediococcus Pentosaceus*	X_6_ (%)	7	10	13	12	9	14	6	15	8	11
SOD Activity	Y(U/mL)	676.14	667.37	653.22	680.00	685.76	664.45	683.65	646.35	659.41	661.27

In the regression equation, R^2^ = 0.999, *p* < 0.01, indicating that the equation was able to accurately predict the best conditions. In summary, it was predicted that the optimal inoculation ratio of FSBJ was 30% for *Z. mobilis*, 5% for L. casei, 13.75% for *L. plantarum*, 31.25% for P. acidilactici, 12.5% for *L. animalis*, and 7.5% for *P. pentosaceus*, while the corresponding SOD activity of this ratio was 733.31 U/mL. According to the above optimal inoculation ratio, the total inoculum of six bacterial strains was maintained at 2 × 10^6^ CFU/mL, while the TSS was 14 °Brix. Then, fermentation was conducted at 37°C for 20 h. The SOD activity of FSBJ measured in three parallel experiments was 703.88 ± 6.37 U/ml, which was better than that achieved by fermentation using only a single strain.

### 3.2. Results of RSM and analysis of variance

It is necessary to investigate the optimal fermentation conditions, as the metabolic mode and metabolic efficiency of microorganisms vary under different conditions, thereby affecting the quality of the products. RSM is a scientific experimental design method. After collecting the data, we used data processing software to fit the equation and predict the effects of various conditions on the response value. Based on the findings obtained from the previous experiments, it was found that the pH and TSS content of SBJ did not change significantly during the fermentation process. To determine the best conditions for the fermentation process, the SOD activity and TFC were used as dual response values to design and optimize the SBJ fermentation conditions.

**FIGURE 2:**
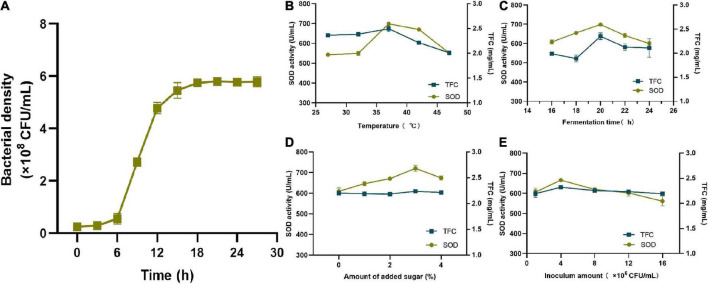
**(A)** Shows the changing trend of lactic acid bacteria density during fermentation; **(B–E)** shows the impact of the temperature, fermentation time, amount of added sugar, and inoculum amount on the SOD activity and TFC of FSBJ.

As shown in Figure 2A, the bacterial growth curve presented an “S” shape, entering the log growth phase at 6 h followed by a plateau at approximately 18 h. Fermentation temperature had a significant impact on SOD activity and the TFC. As shown in Figure 2B, SOD activity reached its peak of 698.94 U/mL at 37°C, and then decreased as the temperature rose. TFC also reached its highest value of 2.49 mg/mL at 37°C and declined rapidly thereafter. The suitable growth temperature of lactic acid bacteria is generally between 30°C and 37°C, and the decrease in both may be due to excessive temperature affecting the bacterial growth. As a result, the optimal fermentation temperature was 37°C. As shown in Figure 2C, SOD activity increased rapidly between 16 h and 20 h, peaked at 698.94 U/mL at 20 h, and then declined. The TFC reached a maximum value of 2.35 mg/mL at 20 h and gradually decreased to 2.11 mg/mL at 24 h. Therefore, it was more reasonable to set the fermentation time to 20 h. Sugar can be used as a carbon source for bacterial growth. As shown in Figure 2D, when the amount of added sugar was 3%, the maximum activity of SOD was 720.78 U/mL and the TFC was 2.24 mg/mL when the amount of added sugar was 3%. As a result, 3% was determined to be the optimal amount of added sugar. As shown in Figure 2E, when the inoculum concentration was 10^6^ CFU/ml, the number of bacteria was insufficient and their metabolic rate of bacteria was slower, which resulted in lower SOD activity and TFC. However, as the inoculum amount increased, so did the values of both indicators. When the inoculum amount was 4 × 10^6^ CFU/mL, SOD activity reached a maximum of 665.91 U/mL, and the TFC reached 2.32 mg/mL. If the amount of inoculation were to exceed 4 × 10^6^ CFU/mL, it may lead to excessive fermentation and a change in the pH of the resulting FSBJ, thus affecting SOD activity and the TFC. Therefore, the optimal range of the inoculum amount was 4 × 10^6^ CFU/mL.

Preliminary experiments revealed that four factors [temperature (A), fermentation time (B), amount of added sugar (C), and inoculum amount (D)] influenced both SOD activity and the TFC. Table 7 shows the Box-Behnken trial design and results. The regression equation models for the response surface are as follows:

$$\begin{array}{cc} {\text{Y}}_{1}\,= & 731.64-3.51\,\text{A}+4.13\,\text{B}-6.97\,\text{C}-8.67\,\text{D}-12.55\,\text{AB}\\ & +6.99\,\text{AC}-10.47\,\text{BC}+11.22\,\text{BD}+6.36\,\text{CD}-33.61\,{\text{A}}^{2}\\ & -24.83\,{\text{B}}^{2}-36.44\,{\text{C}}^{2}-36.79\,{\text{D}}^{2} \end{array}$$

$$\begin{array}{cc} Y_{2}\,= & \,2.49+0.03\,\text{A}+0.02\,\text{C}+0.02\,\text{D}-0.04\,\text{AC}-0.06\,\text{BC}\\ & +0.03\,\text{BD}-0.12\,{\text{A}}^{2}-0.07\,{\text{B}}^{2}-0.04\,{\text{C}}^{2}-0.03\,{\text{D}}^{2} \end{array}$$

**TABLE 7:** Sensory attributes of the fermented sea buckthorn juice products.

Category	Attributes	Score
Appearance	Orange color	0–10
	Clarity	0-10
Flavor	Sea buckthorn flavor	0–10
	Wine bouquet	0–10
	Sour flavor	0–10
Taste	Acid-sugar ratio	0–10
	Sea buckthorn taste	0–10
	Astringency	0–10
	Wine taste	0–10
Overall	Acceptability	0–10
	Purchase intention	0–10

0–3, the characteristics were hard to assess; 4–6, the characteristics could be assessed to some degree; 7–10, the characteristics were easily discernable.

The analysis of variance (ANOVA) for the RSM model is shown in Table 4. The regression model optimized here was highly significant (*p* < 0.01), whereas the lack of fit was not (*p* > 0.05). Meanwhile, the model regression coefficient R^2^ of the SOD activity was 0.9835, while and the model regression coefficient R^2^ of TFC was 0.9598, showing that the model had a good simulation effect and could effectively predict the fermentation parameters of SBJ.

**FIGURE 3:**
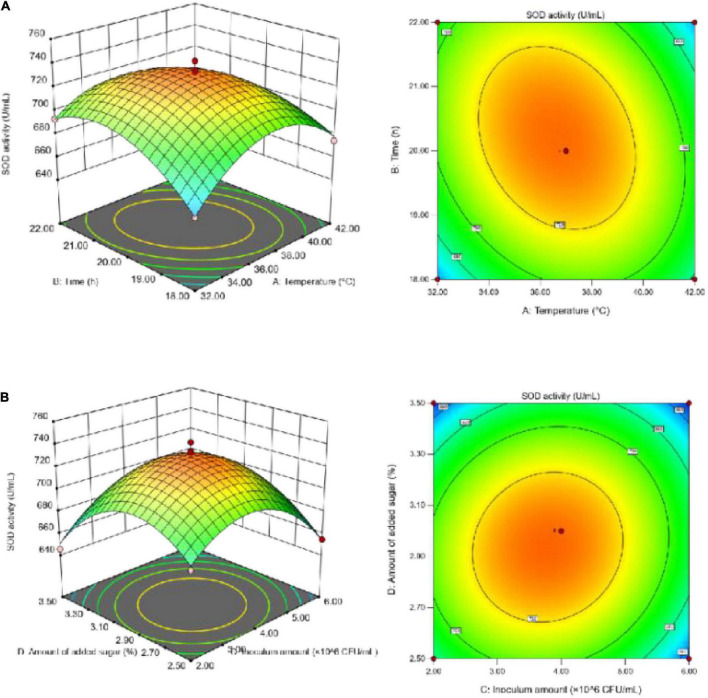
**(A)** Effect of the interaction between fermentation temperature and fermentation time on SOD activity; **(B)** effect of the interaction between inoculum amount and sugar addition on SOD activity.

**FIGURE 4:**
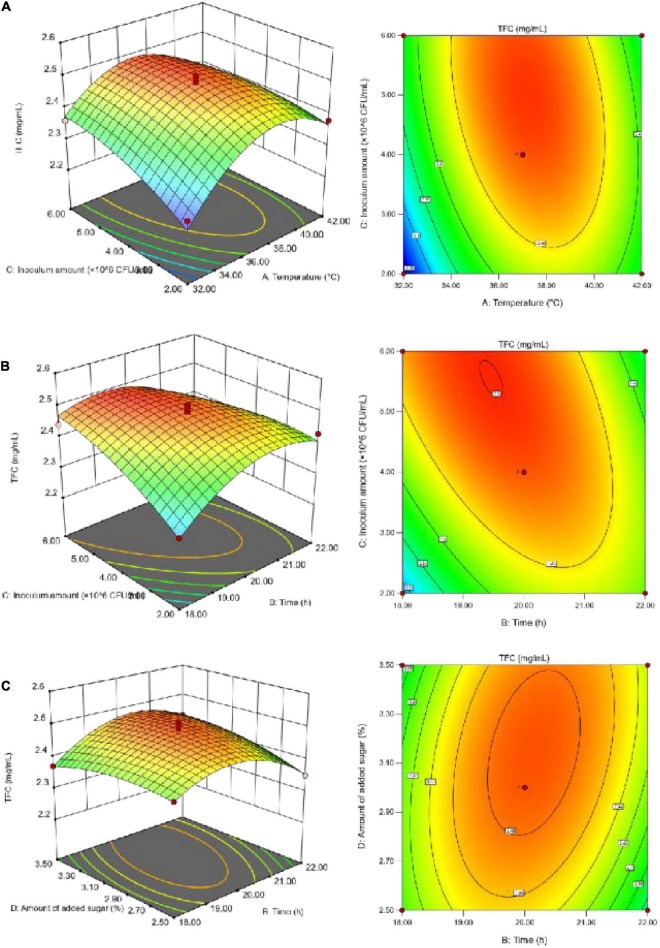
The effect of interaction on the TFC. **(A)** The interaction between temperature and inoculum amount; **(B)** the interaction between fermentation time and inoculum amount; **(C)** the interaction between fermentation time and sugar addition.

To better visualize the significant and positive interaction effects of the fermentation parameters on SOD activity (Figure 3) and the TFC (Figure 4), 3D response surface plots were generated. According to Figure 3, SOD activity increased initially with the increase in each individual factor value until it reached a peak, and then decreased. Sugar addition had the greatest effect on SOD activity. If the temperature was too high, it negatively affected the growth rate of the microorganisms, resulting in a decrease in SOD activity. Due to the instability of SOD, the addition of both a large amount of sugar and large number of inoculated bacteria likely led to the accumulation of more metabolic wastes by microorganisms, thereby resulting in the decline in SOD activity.

According to Figure 4, the TFC increased initially with increasing individual factor values until it reached a peak, and then decreased; the fermentation temperature had the greatest impact on the TFC. With too high a volume of inoculum and amount of added sugar, the microorganisms in SBJ grew vigorously and consumed a large sum of the flavonoids to support their growth and metabolism (27), thus leading to a decrease in the TFC. The Design-Expert 13 software analysis was used to determine the optimal fermentation conditions, as follows: a duration of 20.04 h, temperature of 37.21°C, inoculum concentration of 4.08 × 10^6^ CFU/mL, and final added sugar concentration of sugar addition of 2.98% The predicted values for the FSBJ were 731.44 U/mL of SOD activity and 2.49 mg/mL of the TFC. The optimal fermentation duration and temperature were changed to 20 h and 37°C to simplify the protocol’s operability. Multiple parallel experiments confirmed that SOD activity was 725.44 U/mL and the TFC was 2.38 mg/mL under the optimized conditions. The established model thereby not only improved the SOD activity of the fermented products but also ensures a high TFC.

### 3.3. Antimicrobial activity

**FIGURE 5:**
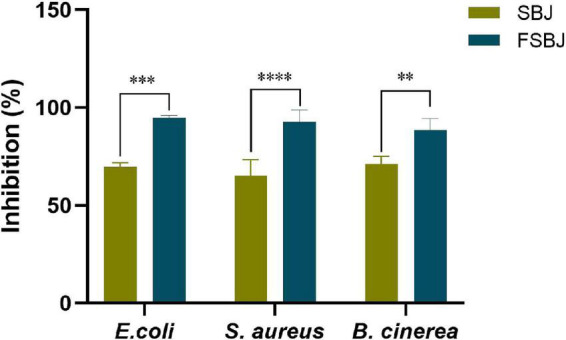
Antibacterial and antifungal activities of FSBJ and SBJ (**p < 0.01, ***p < 0.001, ****p < 0.0001).

The antibacterial effect and antifungal effect of FSBJ and SBJ were evaluated (Figure 5). The results indicated that antimicrobial properties increased during fermentation and that the fermented juice had higher antibacterial properties against *E. coli* in comparison to *S. aureus* and *B. cinerea*. Fermentation has been shown to increase the antibacterial and antifungal activities of fruit juices (28). The findings of this study are consistent with previous research (29). FSBJ had more significant antibacterial and antifungal activities than SBJ, which may be related to the production of organic acids and bacteriostatic proteins during the fermentation (30). Studies have shown that *L. plantarum* can generate fumarate (31), which is an effective inhibitor of *E. coli* (32). Meanwhile, antibacterial activity is also affected by pH. Experiment data showed that SBJ still had bacteriostatic activity, indicating that sea buckthorn originally contained components with bacteriostatic activity, such as polyphenols, flavonoids, and organic acids. After fermentation, the content of these substances increased, which may have led to the observed increase in the antibacterial and antifungal activities of FSBJ.

### 3.4. Antioxidant activity of FSBJ

#### 3.4.1. Effect of SBJ and FSBJ on H_2_O_2_-induced viability in C2C12 cells

**FIGURE 6:**
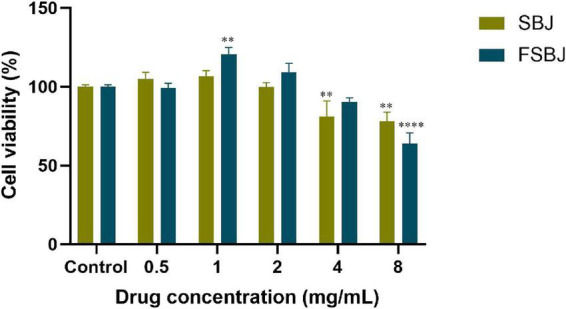
Effects of different concentrations of SBJ and FSBJ on cell survival rates compared to the control group (**p < 0.01, ****p < 0.0001).

To further evaluate the antioxidant activities of SBJ and FSBJ, H_2_O_2_-treated C2C12 cells were used to establish a cell model of oxidative stress. After adding 0, 0.5, 1, 2, 4, or 8 mg/mL of H_2_O_2_ to the medium of C2C12 cells and leaving them for 24 hours, the toxicity-protective effects of SBJ and FSBJ on the cells were measured using the CCK-8 kit, as illustrated in Figure 6. Statistical analysis revealed that the difference in cell viability between the control group and the 0.5 mg/mL FSBJ-treated group was not statistically significant (*p* > 0.05).

Cell survival rates were improved compared to that of the control when treated with 1-2 mg/mL FSBJ. When the FSBJ concentration was 1 mg/mL, cell viability increased by 20.52%. Higher concentrations of FSBJ (4 and 8 mg/mL) significantly reduced cell viability. The results indicate that FSBJ had no toxicity on the C2C12 cells within the concentration range of 0.5-2 mg/mL. Therefore, all subsequent experiments were conducted using these non-toxic concentrations.

**FIGURE 7:**
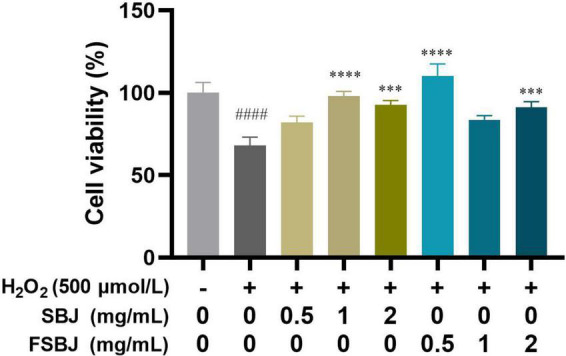
The effects of SBJ and FSBJ on the survival rates of C2C12 cells exposed to 500 μmol/L H_2_O_2_ compared to the control group (no H_2_O_2_, ^####^*p* < 0.001) and damaged group (non-SBJ and FSBJ supplemented, ***p < 0.001, ****p < 0.0001).

To determine the optimal concentration of SBJ and FSBJ for protecting C2C12 cells against H_2_O_2_-induced oxidative stress, cells were pre-treated with 500 μmol/L H_2_O_2_ for 24 h and then treated with 0.5, 1, or 2 mg/mL of either SBJ or FSBJ for 24 h. The rates of cell viability of the SBJ and FSBJ-administered groups were significantly higher than that of the H_2_O_2_-treated group without juice (***p < 0.001, Figure 7). The cell viability of the SBJ-treated group at 1 mg/mL was almost as high as that of the control group, while the cell viability of the 0.5 mg/mL FSBJ-treated group was 10.28% higher than that of the control group. These findings indicate that SBJ and FSBJ can both protect C2C12 cells from oxidative stress, with FSBJ showing a superior effect.

#### 3.4.2. Effect of SBJ and FSBJ on intracellular ROS levels in C2C12 cells

The intracellular ROS level is an important indicator of cellular damage (33) and is also linked to the regulation of multiple signaling pathways (34). To further investigate the protective effect of FSBJ against H_2_O_2_-induced oxidative stress in C2C12 cells, we used flow cytometry with the fluorescent dye DCFH-DA to detect intracellular ROS production.

**FIGURE 8:**
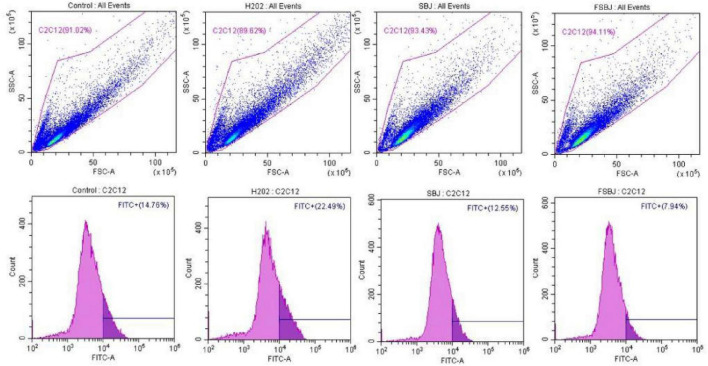
The inhibitory effect of SBJ and FSBJ on H_2_O_2_-induced intracellular ROS production in C2C12 cells.

The results of the flow cytometry analysis are shown in Figure 8. The amount of ROS fluorescence was significantly higher in the H_2_O_2_-treated group compared to the control group, indicating that H_2_O_2_ markedly promoted ROS generation. By contrast, the fluorescence intensity of ROS was significantly lower in the FSBJ-treated group than in the H_2_O_2_-treated group; notably, it was even 2.21% lower than that of the control group. These results imply that FSBJ intervention effectively reduces ROS production induced by H_2_O_2_ and hence contributes a reparative effect on damage caused by cellular oxidative stress.

#### 3.4.3. Effects of FSBJ on antioxidant enzyme activity and MDA contents

The body employs an antioxidant enzyme systems including SOD, GSH-Px, and CAT to defend itself against damage from oxidative stress. These enzymes are endogenous antioxidants and play an important role to keep the dynamic equilibrium of oxidative stress (35). These enzymes have the ability to scavenge free radicals in the body (36), and their activity reflects the body’s antioxidant capability. MDA is a byproduct of lipid peroxidation, and its level both reflects the degree of lipid peroxidation *in vivo* and indirectly reflects the degree of cellular damage (37).

**FIGURE 9:**
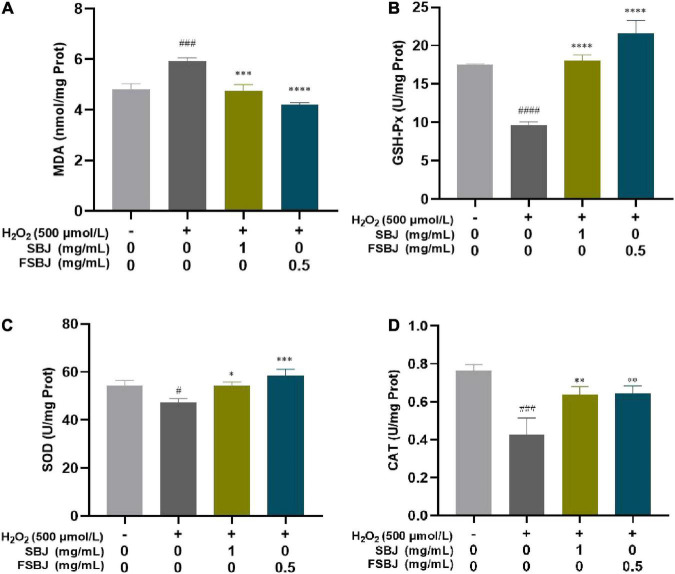
The effect of FSBJ on the oxidative stress indexes of C2C12 cells compared to the control group (no H_2_O_2_, ^#^*p* < 0.05, ^###^*p* < 0.001, and ^####^*p* < 0.0001) and damaged group (non-SBJ and FSBJ-supplemented, *p < 0.05, **p < 0.01, ***p < 0.001, and ****p < 0.0001). **(A)** Effects of FSBJ on MDA contents; **(B)** effects of FSBJ on GSH-Px activity; **(C)** effects of FSBJ on SOD activity; and **(D)** effects of FSBJ on CAT activity.

It can be seen from Figure 9A that the MDA content of C2C12 cells in the damaged group increased significantly compared to that of the control group. With the FSBJ treatment, the intracellular MDA content decreased significantly. These results indicate that FSBJ protected cells against lipid peroxidation caused by ROS, thereby alleviating cellular oxidative damage caused by the free radicals. The activities of SOD, CAT, and GSH-Px in the C2C12 cells were significantly lower in the damaged group compared to the control group, whereas the FSBJ treatment significantly increased their activities (Figures 9B–D). These findings imply that FSBJ treatment enhances endogenous antioxidant defense.

## 4. Conclusion

In this study, Xinjiang-cultivated sea buckthorn was obtained and used as a raw material to assess its antioxidant effect following fermentation. Firstly, six bacteria were screened for the mixed fermentation of sea buckthorn juice based on the resulting products’ SOD activity, TFC, and sensory evaluation. Then, the uniform design method was used to determine the optimal inoculum concentration of each strain for the fermentation process. SOD activity and TFC values were used as indicators to optimize the fermentation process of sea buckthorn juice using the RSM. When the number of bacteria inoculated was 4.08 × 10^6^ CFU/mL and the inoculation ratio was 30% *Z. mobilis*, 5% *L. casei*, 13.75% *L. plantarum*, 31.25% *P. acidilactici*, 12.5% *L. animalis*, and 7.5% *P. pentosaceus*, the amount of sugar was 2.98% (w/v), and the SOD activity reached 725.44 U/mL, and the TFC reached 2.38 mg/mL after 20 h of fermentation at 37 °C.

Fermentation of sea buckthorn juice could improve its biological properties such as antifungi activity as well as antibacterial properties. Fermentation has been shown to increase the antioxidant activity of the resulting products, but there have been few studies on the antioxidant activity of fermented sea buckthorn juice. We hence performed cell experiments to investigate this, and the results show that both cell survival and the activity of antioxidant enzymes were significantly increased when FSBJ was used to alleviate the harmful of oxidative stress induced by H_2_O_2_ in C2C12 cells. However, this study only performed a preliminary analysis of the antioxidant activity and effect on oxidative damage of FSBJ in vitro. It still remains unknown how the antioxidant substances in sea buckthorn change during fermentation, as well as the protective mechanism underpinning the antioxidant effect *in vivo* of FSBJ, so these warrant further investigation.

## Data availability statement

The original contributions presented in this study are included in this article/supplementary material, further inquiries can be directed to the corresponding authors.

## Author contributions

XL and LW: conceptualization. XL and ML: methodology. RM: software and supervision. ML and KC: data curation. XL: writing—original draft preparation. LW and ML: writing—review and editing. JY: visualization. AA and LW: project administration. KC: funding acquisition. All authors have read and agreed to the published version of the manuscript.
